# Supplementary material for: Phytochemical and Biological Characteristics of Mexican Chia Seed Oil
Source: Molecules. 2018 Dec 6;23(12):3219. doi: 10.3390/molecules23123219 (PMC6321551; doi:10.3390/molecules23123219)
Supplement: Supplementary file 1 [file molecules-23-03219-s001.pdf]

Supporting information

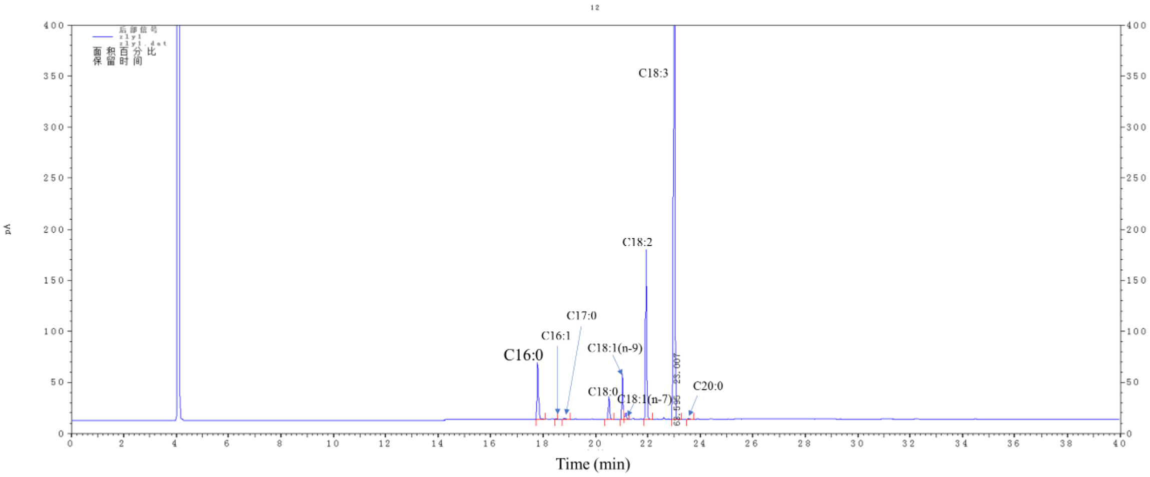


**Figure S1**. HPLC chromatogram of fatty acids or chia seed oils.

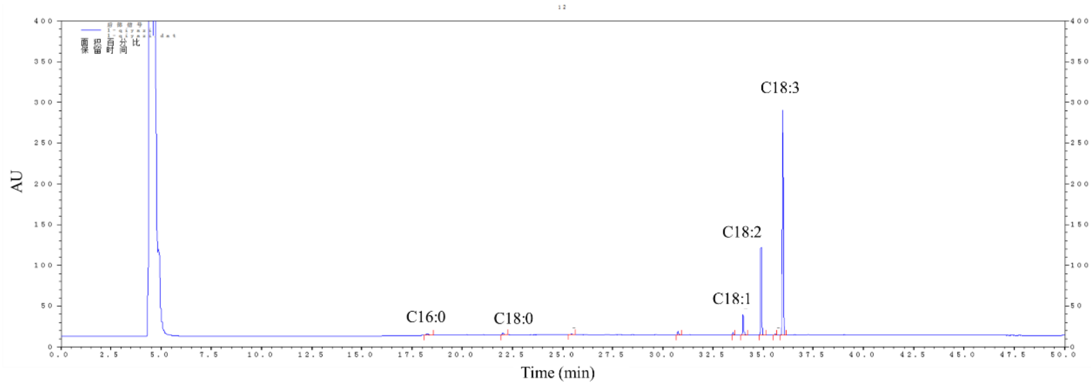


**Figure S2.** HPLC chromatography of sn-2 fatty acids from chia seed oil.

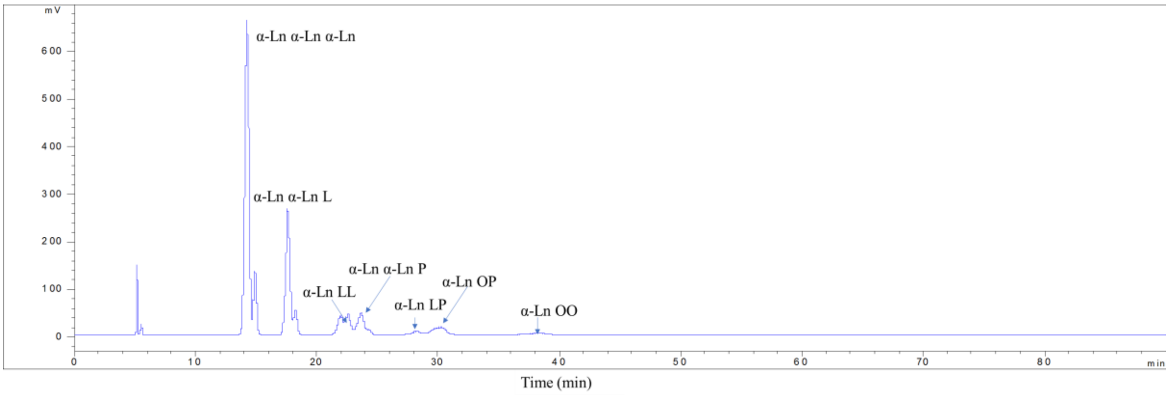


**Figure. S3**. Analysis of triacylglycerols from chia seed oil by HPLC.

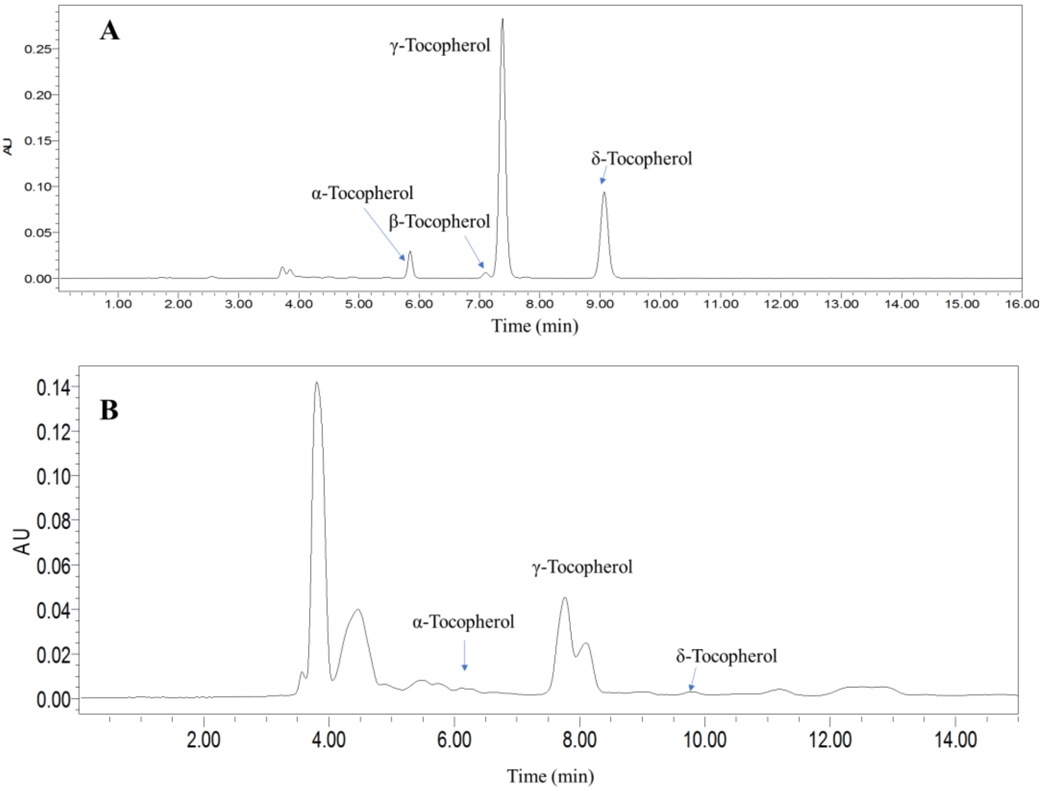


**Figure S4.** HPLC chromatogram of tocopherols in chia seed oil. (A) standards of tocopherols, and (B) tocoperols in chia seed oil.
